# Participants' experiences with the hybrid digital psychotherapy intervention-DIGIT for obesity and distress: A qualitative interview study

**DOI:** 10.1016/j.invent.2026.100997

**Published:** 2026-09-08

**Authors:** Tania Lalgi, Charlotte Michel, Sophie Schulz Genannt Menningmann, Britta Pehlke, Christoph Jansen, Till Hasenberg, Martin Teufel, Alexander Bäuerle, Anita Robitzsch

**Affiliations:** a Clinic for Psychosomatic Medicine and Psychotherapy, LVR-University Hospital Essen, University of Duisburg-Essen, Essen, Germany; b Center for Translational Neuro- and Behavioral Sciences (C-TNBS), University of Duisburg-Essen, Essen, Germany; c Sana Obesity Center North Rhine-Westphalia, Remscheid/Düsseldorf, Germany; d Faculty of Health, University of Witten/Herdecke, Witten, Germany

**Keywords:** Digital therapy, Blended, Obesity, Thematic analysis, Mental healthcare, Psychotherapy

## Abstract

**Background:**

Obesity is a major burden for the individual affected and poses major challenges to healthcare systems worldwide. The intricate link between obesity and mental health disorders raises the need for innovative interventions that address the multitude of barriers hindering individuals with obesity from receiving adequate mental health care.

**Objectives:**

This qualitative study explored participants' experiences of the digital inpatient-like and interprofessional DIGIT intervention, focusing on acceptability, perceived benefits and challenges, as well as perceived changes associated with participation.

**Methods:**

Fourteen semi-structured interviews were conducted in German following participants' completion of the DIGIT intervention. The data were analyzed using reflexive thematic analysis, guided by the six-phase framework of Braun and Clarke. Two researchers initially coded the data, while a third researcher contributed to the iterative process and refinement of themes. Team discussions were used to enhance reflexivity and interpretive depth rather than to establish coding reliability.

**Results:**

The study identified four categories: (1) user experience, (2) participant needs, (3) perceived benefits, and (4) facilitated access to treatment. Participants reported high levels of acceptance and satisfaction with the treatment. Moreover, participants reported subjective symptom relief, improved disease-related coping, and an overarching activating effect that they associated with their participation in DIGIT. Furthermore, participants reported a reduction in barriers to accessing this specific DIGIT intervention compared to conventional treatment options.

**Conclusion:**

This study highlights the advantages of digital inpatient-like therapy concepts. Patients' high acceptance of and satisfaction with the inpatient-like therapy is an important facilitator for future implementation of innovative treatment concepts. Treatment concepts like DIGIT can contribute to providing individualized therapy options and closing care gaps.

## Highlights

• A digital, hybrid inpatient-like psychotherapy (DIGIT) was explored qualitatively among individuals with obesity and psychological distress.
• Participants described a strong therapeutic alliance despite the digital format, especially in individual online sessions.
• Perceived facilitators included flexible access, therapist competence, and structure; barriers wereduration and no follow-up.
• DIGIT may offer an acceptable and accessible hybrid treatment option for individuals with obesity and psychological distress.

## 1 Introduction

Obesity is defined as a body mass index (BMI) ≥ 30 kg/m^2^ and is classified as a complex chronic disease (World Health Organization, 2024). From 1990 to 2022, the global prevalence of obesity among adults has more than doubled (Koliaki et al., 2023; World Health Organization, 2024). Despite the flattening of obesity prevalence in industrialized countries, a significant increase in the number of people with severe obesity in high-income populations (BMI ≥ 40 kg/m^2^) is predicted between 2020 and 2035, leading to an estimated increase in prevalence from 10 to 20% (Koliaki et al., 2023).

Obesity is associated with an increased risk of various physical conditions, including type 2 diabetes, overall higher cardiovascular mortality, and various types of cancer (Koliaki et al., 2023; Sarwer and Polonsky, 2016; World Health Organization, 2024). Not only is obesity associated with an increased risk of physical conditions but also with a heightened risk of comorbid mental disorders. Recent systematic reviews confirm that electronic health (eHealth) programs addressing both physical health and psychological needs are most effective in reducing depressive symptoms in people with obesity (Kocol et al., 2024). This link is partially disregarded in the primary unifaceted physical treatment of obesity. The current demand for resource-intensive inpatient mental and physical treatment for people with obesity cannot be met by the healthcare system (Dandgey and Patten, 2023; Rajan and Menon, 2017).

This study aims to address these limitations by examining the digital inpatient-like, interprofessional treatment program, DIGIT, that is accessible to individuals with obesity. One way to make healthcare more accessible could be the use of communication and information technologies (e.g., video consultation, medical apps). In contrast to standard telehealth psychotherapy or single-component online interventions, DIGIT provides a holistic, coordinated six-week program that integrates individual psychotherapy, group psychotherapy, nutritional counseling, digital therapeutic materials, and selected in-person appointments. DIGIT is conceptualized as “inpatient-like” because it combines the structured, intensive, and multimodal organization of inpatient care within a predominantly digital, outpatient-based treatment format. Digital interventions enable a location-independent, flexible, low-threshold, and potentially resource-saving treatment to surmount various barriers to face-to-face therapy (Schmidt-Hantke et al., 2021). Previous research has proven the effectiveness of cognitive behavioral therapy (CBT) telehealth interventions within a multidisciplinary protocol. Such interventions have been associated with facilitating weight loss, improve the quality of life, decrease anxiety, and support adherence to healthcare recommendations (Castelnuovo et al., 2017). Digital health interventions in weight management show promising results in the care of people with obesity (Kupila et al., 2023). Nonetheless, in-person consultations might be necessary to enable a comprehensive treatment setting. Hybrid treatment concepts enable the inclusion of both digital and in-person treatment elements. Thus, hybrid therapeutic interventions combine the advantages of digital and on-site interventions and can thereby target a complex patient group while providing a high-quality treatment (Galin, 2024; Haleem et al., 2021).

The qualitative study aimed to gain an in-depth understanding of the patients' experiences with DIGIT. A particular focus was on acceptability, perceived benefits and challenges, and alignment with patients' individual needs. Specifically, the qualitative research questions were: (1) How do participants experience DIGIT? (2) How do participants perceive the individual and group-based therapeutic components? (3) Which aspects of the intervention do participants perceive as beneficial or challenging? (4) Which factors facilitated or hindered participants' engagement with the intervention?

## 2 Methods

### 2.1 Study design

Building on existing findings, qualitative research offers deeper insight into the mechanisms underlying change processes within specific patient groups, as well as their individual needs and expectations. This approach provides a more comprehensive understanding of how participants experience the intervention and which processes they perceive as relevant (Lutz and Hill, 2009; Truijens, 2017). It allows for the exploration of nuanced aspects such as the therapeutic alliance in hybrid treatment settings, patient acceptance, and the participants' lived experience. These insights help refine current interventions, inform future program development, and contextualize outcomes observed in quantitative research (Creswell et al., 2007; Sarwer and Polonsky, 2016).

This qualitative study was conducted following approval by the Ethics Committee of the Medical Faculty of the University of Duisburg-Essen (23-11642-B0). A reflexive thematic analysis (Braun and Clarke, 2006, Braun and Clarke, 2023) was employed to explore the subjective experiences of patients who completed the DIGIT intervention. The study adhered to the COREQ reporting standards (Tong et al., 2007; see Appendix Table A1) and quality criteria proposed by Yardley (2000). To reduce the potential influence of prior treatment-related relationships, data collection as well as analysis were conducted by researchers who were not involved in providing the intervention.

### 2.2 Intervention overview — ‘DIGIT’

**Fig. 1:**
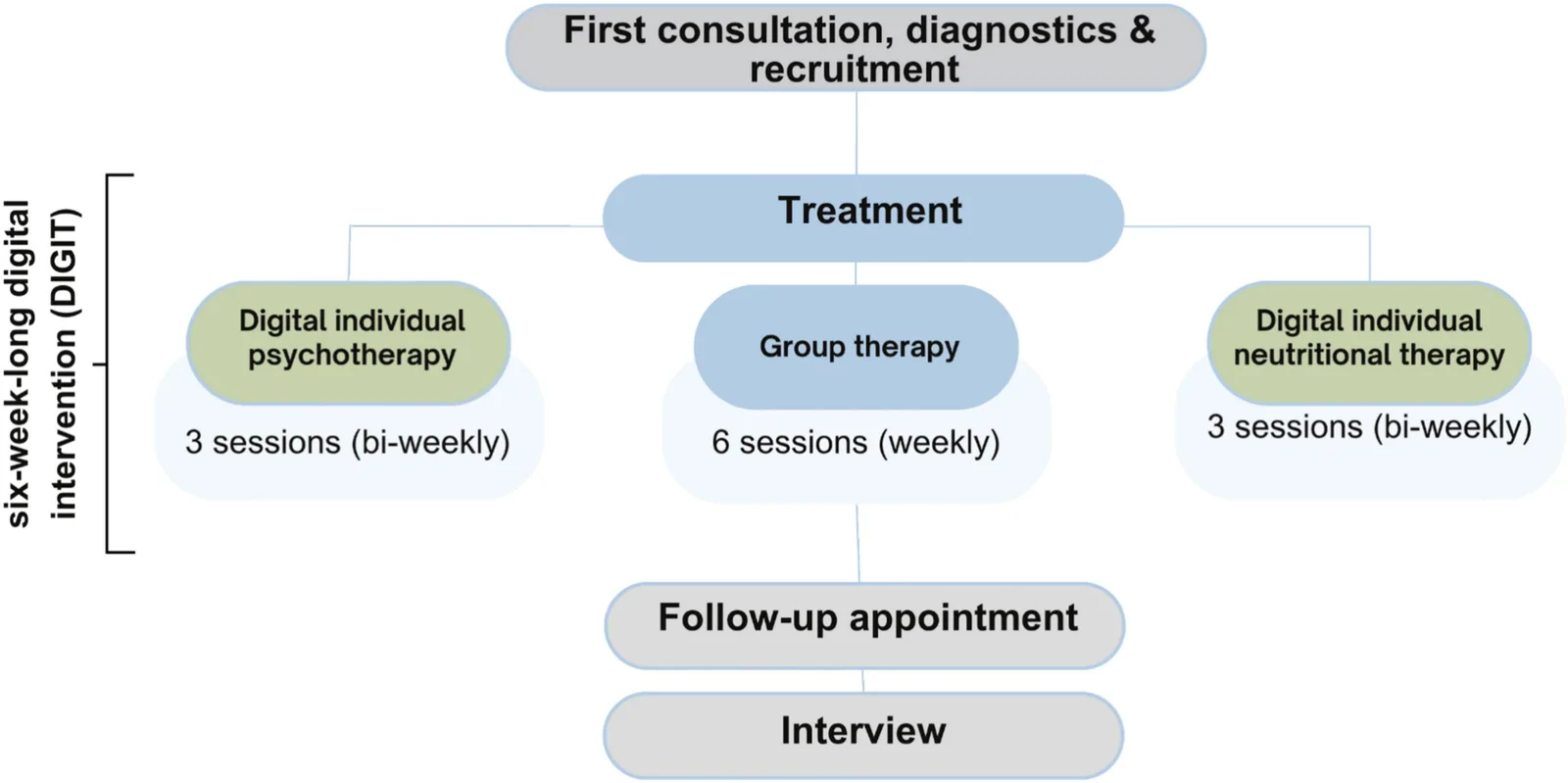
Flow chart illustrating participant recruitment, intervention structure, and post-intervention interviews. *Note*. The different colors represent the format of the intervention components (grey = onsite; blue = hybrid (both online and onsite sessions); green = online). (For interpretation of the references to color in this figure legend, the reader is referred to the web version of this article.)

The DIGIT intervention was developed to support individuals with obesity and distress by reducing psychological strain and improving disease-related coping. In designing DIGIT, variables (e.g., performance expectancy, usability, social influence, and digital self-efficacy) known to predict e-mental health acceptance were used (Rentrop et al., 2022). Over six weeks, DIGIT combined elements of cognitive behavioral therapy (CBT), mindfulness-based stress reduction (MBSR), and acceptance and commitment therapy (ACT). The program included weekly 75-min group sessions provided by a psychologist with a group size of six in the first and eight patients in the second intervention cycle. Additionally, 50-min sessions with bi-weekly individual psychotherapy were provided by either a physician specialized in psychosomatic medicine and psychotherapy or a psychologist; the nutritional consultations were provided by a nutrition scientist. Intervention fidelity was supported by weekly supervision of the multiprofessional team by the senior physician. Additionally, downloadable digital worksheets integrated into the six modules (one/week) were hosted on a dedicated website developed by the in-house web designer in compliance with applicable data security standards. The intervention was delivered via the samedi and webprax platforms (samedi GmbH, 2026; Healthy Projects GmbH, 2026), which process personal data in accordance with the requirements of the General Data Protection Regulation (GDPR; DSGVO). A technical check was offered prior to the intervention, with technical support available throughout the intervention. Three in-person appointments were integrated into the otherwise digital framework: the initial and final individual sessions, as well as the third group session, to obtain a more comprehensive clinical impression of the patient and address any upcoming questions or concerns. In the first group session, emergency contacts and a crisis plan were developed with the patients. These on-site appointments also facilitated medical assessments such as weight measurement and bioelectrical impedance analysis. Nutritional counseling aimed to build realistic expectations regarding eating behaviors, dietary adjustments, and long-term lifestyle changes. The bi-weekly individualized psychotherapy sessions focused on individual goal setting, identifying stressors, and establishing coping strategies. Group sessions addressed psychoeducational topics including comorbidities, vulnerabilities, (self-) stigmatization, and relapse prevention (see Fig. 1 for the intervention flow chart and appendix table A3 for a session-by-session table).

### 2.3 Reflexivity and analytic rigor

Two researchers initially coded the data independently. A third researcher supported the development and refinement of categories and themes. Differences in interpretation were discussed within the research team. As part of an iterative process, the research team met regularly to discuss differing interpretations, reflect on different interpretations, and collaboratively deepen their understanding of the data based on the transcript material.

### 2.4 Participant recruitment and inclusion

A total of 14 participants were recruited between January and February 2024 through the outpatient obesity consultation service of the [blinded for peer review]. Potential exclusion criteria were assessed prior to participation using an in-house clinical interview and standardized questionnaires. These included acute suicidality, severe psychiatric symptoms requiring immediate treatment, severe cognitive impairment, and medical or psychological conditions that could interfere with participation in the intervention. Participants were recruited using consecutive sampling. All patients meeting the inclusion criteria were informed about DIGIT. Interested individuals received study information via email or phone. During the initial recruitment period, before the intervention started, some participants withdrew (e.g. due to reported time constraints). Inclusion criteria were: (1) age ≥ 18, (2) fluent in German, (3) BMI ≥ 40 kg/m^2^, (4) significant self-reported distress, (5) no concurrent psychotherapy, (6) access to a digital device with a stable Internet connection, and (7) written informed consent. The inclusion criteria applied both to participation in DIGIT and to inclusion in the qualitative study. All participants had completed the DIGIT program before receiving the invitation to the interview. All individual sessions and at least five out of six group sessions had to be completed to be eligible to participate in the interviews. All participants completed the DIGIT intervention and the subsequent qualitative interview, resulting in no dropouts.

### 2.5 Semi-structured interview

The interview guide (see Appendix Table A2) was based on three validated instruments: the Client Change Interview (CCI; Elliott et al., 2001), the Health IT Usability Evaluation Scale (Health ITUES; Brown et al., 2013), and the German version of the Working Alliance Inventory — Short Revised (WAI-SR; Wilmers et al., 2008). The semi-structured nature of the conducted interviews was chosen to support consistency and completeness while exploring the unique experiences of the participants (Bryman et al., 2012; Myers and Newman, 2007). Additional self-constructed items were included to address expectations, therapeutic experiences, and digital usability (see Appendix Table A2). The guide was developed collaboratively by experts in obesity treatment, psychotherapy, digital health, and qualitative research.

### 2.6 Interview procedure

All semi-structured interviews were carried out in person at the premises of the [blinded for peer review]. Interviews were conducted a mean of 8.71 days (SD = 8.03; range = 0–25) after completion of the intervention. In Cycle 1, interviews were conducted a mean of 11.67 days (SD = 6.71; range = 0–19) after intervention completion, compared with 6.50 days (SD = 8.64; range = 0–25) in Cycle 2. Each participant provided written informed consent and agreed to audio recording. The participants received neither a reward nor compensation for possible expenses. No third party was present during the interviews. Interviews were held in German and recorded on a password-protected device. The interviewer was a clinically trained psychologist undergoing postgraduate psychotherapy training, with prior experience in qualitative research. The interviewer was not part of the treatment team and had no therapeutic relationship with the participants. Transcripts were produced verbatim using f4x transcription software (audiotranskription, 2024), with all personal data pseudonymized or removed during transcription. Quotations presented in the manuscript were translated from German into English for publication and reviewed for semantic equivalence by a bilingual research-team member.

### 2.7 Data analysis

The examination and coding of the transcripts were carried out in line with the guidelines by Braun and Clarke, 2006, Braun and Clarke, 2023. A combined deductive–inductive coding approach was applied. Initial themes were partly theory-driven (e.g., therapeutic alliance) and partly derived from participants' narratives. This hybrid approach allows both structured analysis and openness to unexpected patterns in the data (Braun and Clarke, 2023; Fereday and Muir-Cochrane, 2006). Subsequently, the text analysis was applied with the software for computer-aided data analysis MAXQDA (VERBI Software, 2021). The analysis was an iterative approach, which followed the following six steps for reflexive thematic analysis by Braun and Clarke: (1) Firstly, two coders explored and got familiar with the data. (2) Afterward, both coders performed an independent open-coding phase and established initial themes and possible subthemes. (3) During the third phase, multiple feedback loops between both coders resulted in significant themes. (4) These were further discussed with a third coder, (5) which resulted in a thematic map consisting of ten themes and fifteen subthemes. Research-team discussions served to enhance reflexivity and interpretive depth rather than to establish intercoder agreement. In the final phase, the results were written up in a coherent narrative.

## 3 Results

A total of 14 one-on-one interviews (M = 22 min 10 s; SD = 6 min 44 s; range = 15–32 min) were conducted in this qualitative study. The sample consisted predominantly of women (92.8%; n = 13) and one male participant. All participants reported having obtained a school degree. Table 1 provides an overview of the sociodemographic and medical data of the participants. For the physical diseases and mental disorders, obtained with prior medical records and through questionnaires and a diagnostic interview, multiple selections were possible.

**Table 1:** Sociodemographic characteristics of participants; *Note*. N = 14.

Characteristic	Values	
	N	(%)
Age range (years)		
30–39	3	(21.4)
40–49	5	(35.7)
50–59	5	(35.7)
70–79	1	(7.1)
*Mean*	*48.4*	
Median	47	
*SD*	*11.2*	
IQR	42.2–53.0	
Age range	31–76	
BMI (kg/m^2^)		
40–50	10	(71.4)
50–60	3	(21.4)
60–70	1	(7.1)
*Mean*	*45.3*	
*SD*	*4.3*	
Median	44.1	
IQR	42.3–48.8	
Weight (kg)		
Mean	126.4	
Median	116.6	
SD	21.3	
IQR	112.9–141.0	
Occupational status		
Employed	9	(64.3)
Not employed	3	(21.4)
Retired	2	(14.3)
Distance from the clinic to participants' residences		
<25 km	10	(71.4)
>25–<50 km	2	(14.3)
>50–<75 km	2	(14.3)
Physical diseases		
Hypertension	5	(35.7)
Hypothyroidism	4	(28.6)
Type 2 diabetes mellitus	4	(28.6)
Asthma	1	(7.1)
Metabolic Dysfunction-Associated Steatotic Liver Disease (MASLD)	1	(7.1)
Lipedema	1	(7.1)
Obstructive sleep apnea	2	(14.3)
Hashimoto's disease	2	(14.3)
Mental disorders		
Mild depression	2	(14.3)
Major depressive disorder	6	(42.9)
Unspecified eating disorder	3	(21.4)
Atypical bulimia nervosa	1	(7.1)
Anxiety disorder	2	(14.3)
Posttraumatic stress disorder	1	(7.1)

### 3.1 Theme classifications

**Fig. 2:**
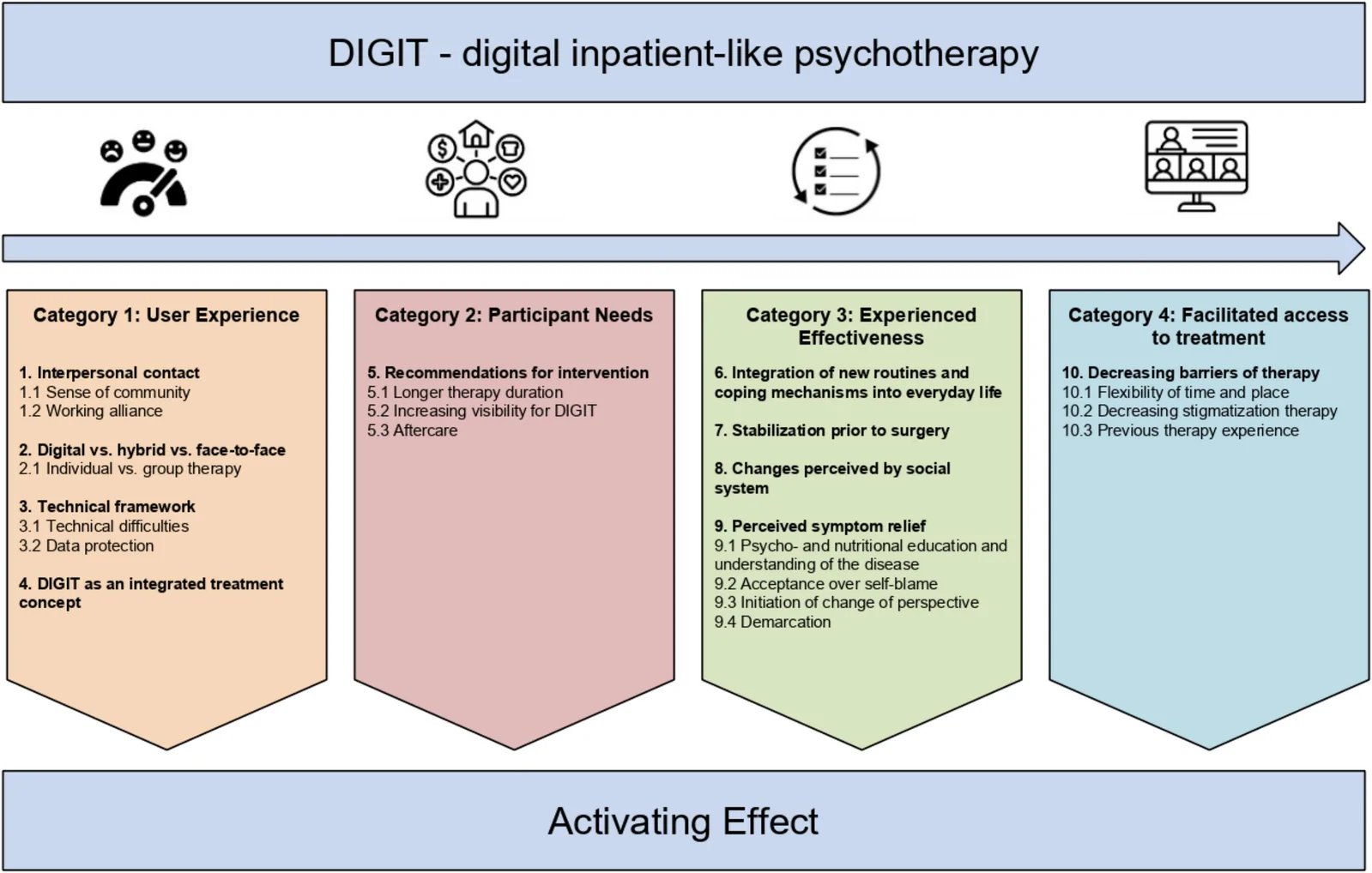
Final codebook structure. Graphic display of the overarching categories, themes, and subthemes identified during the data analysis process. The term “activating effect” describes an overarching finding that was identified across, rather than within, the observed categories.

The finalized codebook, which had been developed following an iterative process, is shown in Fig. 2. The categories “*user experience*” and “*participants' needs*” were derived deductively based on the preexisting categories of the used questionnaires. Additionally, two more categories, namely “*perceived benefits*” and “facilitated access to treatment,” were added inductively during the open-coding phase. Line-by-line coding generated codes that were subsequently grouped to ten themes and a total of fifteen relevant subthemes.

Ten out of 14 participants identified the overarching “activating effect” of DIGIT. They explained that interacting with other patients and the progress of the individual therapy sessions helped them to open up and engage with others socially as well as being my active physically. One participant reflected on this transformation, stating, “*I used to sit in my shell a lot (...) and that has improved*” (Interviewee D), using the metaphor of a snail retreating into its shell to describe social withdrawal. Other participants expressed “*that I go out more*” (Interviewee A); “*that you can go out instead of eating*” (Interviewee F), or “*So maybe I'm a little more active and positive*” (Interviewee E).

#### 3.1.1 Category 1: User experience

##### 3.1.1.1 Theme 1: Interpersonal contact

This theme is based on the two main forms of interpersonal contact forms, namely the group therapy with other patients, as well as the working alliance between the therapists and the patient. Overall, the participants described an accepting, tolerant, and judgment-free space within the patient group and with the therapists, “*Not only the therapist, but also the other group members. I really had the feeling that I could say everything I felt and thought*” (Interviewee D).

###### 3.1.1.1.1 Subtheme 1.1. Sense of community

Two participants expressed that they felt anxious about the group meetings. Moreover, one participant reported that she did not enjoy the group as much because many discussions revolved around topics that were not personally relevant, such as bariatric surgery, which made it difficult to engage in the conversations. When asked about their experience with the other patients, the majority of participants (11/14) reported having felt a sense of connectedness and community with the other patients. All participants experienced the group meetings as supportive because of the shared obesity disease and similar experiences “*(…) you feel somehow connected because we all have the same problem, but everyone has a different approach and that's really supportive.*” (Interviewee A). Some reported being able to learn from the practical examples other people shared during the group meetings, “I can relate to it much better when I hear about what others have experienced. *(…) I take a lot from group therapy*”. (Interviewee A) Some participants also kept in touch via social networks to support each other and share information: “*We have a WhatsApp group. We always exchanged a bit of information with each other there*” (Interviewee L).

###### 3.1.1.1.2 Subtheme 1.2. Working alliance

The alliance with the therapist was reported as supportive and appreciative, where the participants felt seen and validated. This made patients feel at ease, as some were anxious before starting DIGIT. Two patients drew a comparison to prior experiences within the healthcare sector “*People are valued (…) you are not just a number where you go in and then go out*” (Interviewee C). Especially the one-on-one meetings were described as de-tabooing, where topics were discussed that are experienced as being stigmatized or not understood and that could not be addressed in other settings (e.g. family, friends) “*when you can just talk without having to think about it and without being afraid to say it, (...) I found it very helpful*”; “*I believe that we were able to say a lot of things that perhaps no one else in our social environment would understand.*” (Interviewee B). All participants reported defining and working on individual therapy goals with their therapists “*At the beginning, I was asked what my goals were, what I expected and then it was fulfilled and addressed right to the end.*” (Interviewee C).

##### 3.1.1.2 Theme 2: Digital vs. hybrid vs. face-to-face

This theme is centered around the acceptance of the different therapy formats that the participants experienced during the intervention. All participants reported a high acceptance of the digital therapy components. Four out of 14 participants reported that there was no difference between digital and face-to-face therapy, as long as the other person was visible via video. “*For me, it didn't make that much of a difference (...) If it had all been without a camera, I wouldn't have liked it that much. But with the camera, I thought it was great.*”; “*I generally find it just as personal when it takes place with a picture*” (Interviewee B). Over half of the participants (8/14) also recognized the hybrid format as positive, where they got to know the group in person and then continued in an online format. In this context, one participant reflected: “*Sometimes it's easier to be with the people in an online program like this than to see everyone in real life next to each other — it often distracts from the topic. Then you look at your shoes or their handbag.*” (Interviewee J). Two participants reported that despite digital therapy having many advantages, in-person therapy would be perceived as advantageous “*Of course, in-person is always better*” (Interviewee A). Moreover, all participants reported that they would be open to participating in an online therapy format in the future.

###### 3.1.1.2.1 Subtheme 2.1: Individual vs. group therapy

Participants reported a difference between digital group therapy and digital individual therapy. Digital group therapy was described as less personal and easier to be passive compared to one-on-one therapy sessions “*Online, you always have a bit of distance. When you're in group therapy, there is a bit more distance, (…) but when you're in individual therapy, it's not like that, because then you're also face-to-face, so to speak.*” (Interviewee A).

##### 3.1.1.3 Theme 3: Technical framework

The technical framework of DIGIT encompassed a website with the necessary information and worksheets that participants were able to access. “*The website was great, as was the communication via e-mail. You always had new information and help straight away.*” (Interviewee A). The video consultations were held via a certified provider. Overall, the technical framework was reported to be easily accessible “*The experience I have now had with digital technology is that it's not rocket science (…)*”, besides the encountered technical difficulties” (Interviewee D).

###### 3.1.1.3.1 Subtheme 3.1: Technical difficulties

All participants reported encountering technical difficulties with the video consultation website, which was experienced as hindering the overall user experience of DIGIT. One group session also had to be rescheduled because of it. “*So, we had some problems logging into the first provider (…) But then we were able to communicate via email to figure out how to move forward*” (Interviewee E). Multiple participants explained that communication of the source of problems and offering solutions (e.g., trying another video consultation provider, catch-up dates) was necessary. “*It was a pity that the online session didn't always work so well because of the provider. But that was later solved by another provider, so it was great. That's why I can't complain about it now*” (Interviewee A) “*Most of it actually worked perfectly. And if a program crashed during a group meeting, the problem was solved very quickly*” (Interviewee H).

###### 3.1.1.3.2 Subtheme 3.2: Data protection

During the interviews, only one participant reported worries about data protection in the past. Due to the open communication about the different service providers for the video consultations and because DIGIT was developed by a healthcare provider, these factors made her feel safe.

##### 3.1.1.4 Theme 4: DIGIT as an integrated treatment concept

All participants reported that all parts of the intervention, namely group therapy, individual nutritional- and psychotherapy, and digital worksheets completed each other, which three participants called an “*all-round package*” (Interviewee B) “*(…) because this digital clinic was such an all-round package for me (…) the nutritional advice and the psychological advice complemented each other so wonderfully. Yes, it's a good, a very good concept*” (Interviewee D); “*I think both are important, because nutritional therapy focuses more explicitly on food, but still has a bit of a psychological perspective or psychological background. But the psychological aspect is then tailored to you and not just to the food, but to your state of mind. And that complements each other really well*” (Interviewee A). Moreover, all participants reported that it was easy to motivate themselves to participate in DIGIT after the first session and that there were no hurdles to joining and completing DIGIT.

In addition, 8 out of 14 participants prepared for bariatric surgery. Of which two compared the surgery preparation program to DIGIT. Even though the pre-surgery preparation consists of six nutritional consultations compared to only three individual nutritional therapy sessions in DIGIT, participants described the three nutritional therapy sessions as more beneficial. “*But I was very positively surprised by the three sessions with the nutritional scientist because I found the session with the dietician from the MMK* (name of the surgery preparation program) *very tough (…) the session with the nutritionist more informative, more lively and more positive (…) actually also more professionally competent.*” (Interviewee E). The content of DIGIT was described as intense but building on each other and covering all relevant topics. Moreover, two participants reported liking the concept of digital worksheets, which could be accessed after the treatment and used in the format of their choice (e.g., print, digitally).

#### 3.1.2 Category 2: Participant needs

##### 3.1.2.1 Theme 5: Recommendations for intervention

Due to the different applied weight loss methods (lifestyle change vs. surgical procedure), individuals reported the need for personalized pre-and post-surgery content, which was accounted for in the individual therapy but could not be addressed in depth in the group therapy due to the heterogeneous nature of the group. “*Specifically, preparation for the operation (…) perhaps for the protein phase would give you a little more inspiration as to what you can cook.*” (Interviewee A) “*That's the specialization. (…) the doctor or whoever is there in the hospital, there's a protein phase, how long is it and stories like that, that some do one, others don't do any, others supposedly must do a liquid phase for two weeks*” (Interviewee E). Therefore, some participants expressed interest in other therapy offerings, such as post-operative group therapy. Two out of 14 participants expressed the wish to integrate interactive elements into the intervention in the future. One participant, for example, suggested implementing role plays: “*Yes, like a role play. I think I would really like that — just to try out how I can position myself when certain accusations or inappropriate questions come up. Just to practice how to handle such situations*” (Interviewee G). Another suggestion referred to the idea of cooking and eating together as part of the program: “*Yes, maybe something where you sit down together somewhere and cook and eat. That would be nice*” (Interviewee J).

###### 3.1.2.1.1 Subtheme 5.1: Longer therapy duration

Eight out of 14 participants reported benefits and their willingness to continue DIGIT beyond the six weeks. If there were follow-up treatment options, I would also like to continue taking part in digital form” (Interviewee D). “*I would also have no problem continuing to do it afterward. So, for me, it only had advantages*” (Interviewee B). “*I think you could discuss every topic we discussed (...) maybe not just talk about it for an hour, but maybe two, just to have a bit more context*” (Interviewee A). However, participants reported substantial perceived improvements during the six-week treatment period “I am delighted that there is a form of support for me that has achieved so much for me in a relatively short time” (Interviewee D).

###### 3.1.2.1.2 Subtheme 5.2: Increase visibility for DIGIT

Overall, all participants reported that they would or already recommend DIGIT to people with obesity who may have comorbid distress “*I would really recommend it to everyone (….) because it's simply a great opportunity to focus on yourself again and look at “what do I need?*” (Interviewee B). Some participants reported that there should be more public awareness of interventions like DIGIT. One participant stated, “*if there was a bit more openness or more of a platform so that it could reach more people*” (Interviewee A). Another participant specifically emphasized the lack of online presence of the program. “*I think the online presence could be bigger because as I said, I also told others about it and they said: I looked it up online, but I couldn't find anything. Where is it? What is it? I couldn't find anything. And I said: Yes, I couldn't find anything either*” (Interviewee H).

###### 3.1.2.1.3 Subtheme 5.3 Aftercare

This subtheme shows that six out of fourteen participants expressed a clear desire for structured follow-up after the end of the DIGIT intervention. Suggestions included access to additional materials or a mobile application for further guidance and information. One participant explained: “*Yes. And I would've liked to go into some things in more detail. Or maybe to have something like an app where you can go back and read things again*” (Interviewee G). Several participants also emphasized the desire for a follow-up meeting or reunion a few months after the intervention to reflect on their progress and to reconnect with others: *aybe something like a follow-up clinic, six months or so later, two weeks of group sessions again where we can give each other feedback and share experiences*” (Interviewee H). “*I don't know exactly when, but in general I would like to meet again after a few months — just to see how everyone is doing and what paths they have taken. I think that would be nice*” (Interviewee M).

#### 3.1.3 Category 3: Perceived benefits

##### 3.1.3.1 Theme 6: Integration of new routines and coping mechanisms into everyday life

All participants reported introducing new routines during DIGIT and maintaining these (e.g., mindfulness exercises, eating patterns, cooking, sports, weekly planning, gratitude, and food journaling) after the treatment. The participants attributed the integration into their everyday lives potentially to their participation in DIGIT. Together with specific stress and disease coping strategies, participants reasoned that DIGIT helped them to decrease everyday stress with adaptive coping behaviors. “*Because it's such an emotional eating at some point (…) you realize in which situations you might really feel stressed and think no, that would be the wrong thing to do right now*” (Interviewee B), “*if (…) everyday life takes its toll on you, what you can do — for example, going out instead of eating or something like that, taking more time for yourself*” (Interviewee F). Six participants reported that in nutritional individual therapy, mindfulness exercises helped to adaptively cope with emotional eating. “*And I sometimes still incorporate that, for example, when I say: I've had such a stressful day, I just need to do my 23-minute body scan and that's it*” (Interviewee L).

##### 3.1.3.2 Theme 7: Stabilization prior to surgery

Eight out of 14 participants planned to undergo weight loss surgery at the time of the intervention. Participants described a perceived stabilizing effect of DIGIT before surgery. “*I was positively surprised because it stabilized me, and I am now stronger (…) I'm in a good position/place right now*”; “*My negative expectations were that (…) I wouldn't be able to get through the time before the operation well. Because the food intake has to be regulated. Or that I wouldn't be able to implement the change in diet as I would like. And none of that happened because my negative thoughts were somehow taken away from me by dealing with them*” (Interviewee A). “*Because I have to find some kind of solution, even after the operation. And it's really funny because, actually it can't be that way yet because I haven't had the operation yet, but my sense of taste has already changed*” (Interviewee E). The intervention also provided a sense of security for the period following the surgery, as one participant explained “*Yes, first of all, the fear of the time after the surgery was taken away from me. That really was taken from me. And I have the feeling that there is an open door here that if I ever fall into a hole, I can always call. That gives me the security that I did the right thing*” (Interviewee L).

##### 3.1.3.3 Theme 8: Changes perceived by the social system

Twelve out of 14 participants reported that perceived changes attributed to DIGIT have been observed by their social systems (e.g., family, spouses, friends, co-workers). Even though not all participants informed their social system about their participation in DIGIT, changes have still been noticed “*that I no longer come home from work so irritable, for example*” (Interviewee B); “*yesterday a dear friend of mine told me that I was walking differently, that I was walking more actively (…) she also asked me if I had lost weight. And another friend said to me, “Wow, I can finally do something with you again. We were at the cinema. I think it's great. I was really happy.”, They said: “Wow, it's really fun talking to you on the phone again”.*” (Interviewee D).

##### 3.1.3.4 Theme 9: Perceived symptom relief

All participants expressed that they experienced subjective symptom relief during and after DIGIT. Different domains have been reported and attributed to either single aspects of the treatment (e.g., nutritional therapy, group therapy, psychotherapy) or intervention in its totality. One participant stated, “*This digital clinic was such an all-round package for me, all my fears were somehow taken into account, and that of course led to a complex result*” (Interviewee D).

###### 3.1.3.4.1 Subtheme 9.1: Psycho- and nutritional education and understanding of the disease

Psychoeducational group sessions and individual therapy enabled the participants to have a better understanding of obesity as a disease and the associated psychological mechanism and comorbid distress “*Through therapy, I understood that the situation I find myself in is not a personal failure, but a combination of several factors. And what helped me, in particular, was the way in which it was a scientific, not so emotionally charged, approach*” (Interviewee D); “*I rediscovered the emergency kit, also the mindfulness exercises, and the self-image*” (Interviewee A). For some participants, the DIGIT intervention also meant being able to put a name to their experience for the first time. “*People always just said: You're not losing weight. I didn't even know that this could be an eating disorder, binge eating. I had never heard of it before. In a way, it gave a name to what I'm experiencing. It's not just my lack of discipline or that I'm failing, there's more behind it*” (Interviewee L).

###### 3.1.3.4.2 Subtheme 9.2: Acceptance over self-blame

Most participants reported that DIGIT helped to decrease self-stigmatization and self-blame that was experienced. When asked, which topic was the most important, Interviewee A answered: “*Self-esteem! Because I didn't have any (…), I had a very negative attitude towards myself, even though I was actually already doing a lot, but I didn't see it anymore. And it was precisely through the individual therapy that I realized this (…) it was difficult, but it was also good because it's no longer such a problem (…) it's liberating*”; “*I would now describe myself as more self-confident. (…) I believe in myself a bit more (…), and I think it's more solidified now*” (Interviewee B). “*Give yourself time and don't think that everything has to work out now. It has to happen right away, but there will be setbacks both before and after the operation, and that you won't be angry with yourself*” (Interviewee E). One participant reported that this helped to reduce their self-blame: “*I was able to reduce the feeling of shame. It's still there, but not as strong as before, it no longer dominates me*” (Interviewee D).

###### 3.1.3.4.3 Subtheme 9.3: Initiation of change of perspective

Participants reported experiencing a change of perspective during DIGIT. “*It was in a therapy session where I realized that I see some things really, really negatively, which I can't blame other people for. (…) And that's why I always try to assess situations new*”. “*Some things came up that you might not have thought of beforehand, but it helped to change other things. Things that were then brought to your attention (…) And if you keep this in mind, you will notice when you are really stressed and think, no, that would be the wrong thing to do right now*” (Interviewee B). “*Some things came up that you might not have thought of beforehand, but it helped to change other things. Things that were then brought to your attention*” (Interviewee C). Moreover, individuals evaluated the bioelectrical impedance analysis as a valuable tool to see the strength of their body and feel motivated to keep up with their physical activity and change the way they look at their bodies. A change in perspective was also evident among some participants regarding their planned or already completed bariatric surgery. They reflected that a purely physical procedure would not be sufficient to bring about lasting change: “*It also became clear to me that bariatric surgery, like the one I had, is not enough on its own. I also need to look elsewhere at how I deal with certain issues. Yes, that was a real eye-opener for me*” (Interviewee G). Others echoed this thought and emphasized the need to integrate psychological aspects into the overall change process. It became clear that physical changes can often be accompanied and supported by medical interventions, while psychological stress is not amenable to such direct treatment. One interviewee summed this up simply: “*Yes, for example, for me, it was always said that the head doesn't get operated on*” (Interviewee L).

###### 3.1.3.4.4 Subtheme 9.4: Demarcation

Five out of 14 participants reported that during DIGIT, they were better able to recognize their own needs and address their limits. They expressed that they started to say “no” more often, which they had struggled to do before “*that you should just say no a lot more. So there's a lot that I'm taking away from this*” (Interviewee B), “*And now I try to take care of myself first and then my friend*” (Interviewee C), “*That I need to pay more attention to myself*” (Interviewee N).

#### 3.1.4 Category 4: Facilitated access to treatment

##### 3.1.4.1 Theme 10: Decreasing barriers to therapy

Overall, many factors of DIGIT were experienced as lowering the threshold for receiving therapy. Most participants perceived DIGIT as lowering the threshold for entering therapy by allowing them to receive treatment from the familiar environment of their own home. Moreover, participants expressed that interventions like DIGIT could act as a starting point to initiate change and seek further help if necessary. “*It simply gives you the opportunity to exchange ideas with others without having to leave your comfort zone first. And that might also help you to get out of this comfort zone; that if this online therapy is easily accessible, more people would dare to do it because they have their own space (…) I think that's a very good thing*” (Interviewee A).

###### 3.1.4.1.1 Subtheme 10.1: Flexibility of time and place

Participants explained that they see many advantages of a digital therapy offering, mainly the flexibility that comes with it. Especially the independence of location and time was reported to make DIGIT easy to integrate into their everyday lives. “*But online is great because you can combine it with work, because you can do it before work or after work and don't have to drive an extra hour*” (Interviewee A); Moreover, one participant explained that she felt more comfortable with DIGIT because of its distance to her home town, she explained that it helps to open up, as well as helps to make the group more diverse and creating unique input “*You don't have these people living right next door to you, which I personally think is an advantage because you can open up differently*” (Interviewee B).

###### 3.1.4.1.2 Subtheme 10.2 Decreasing stigmatization of therapy

Due to lowering the threshold through the hybrid setting, individuals experienced DIGIT as more accessible than regular therapy and changed their perspective of the reported negative connotation of the word “therapy”. “*(…) It is the idea of therapy, which always sounds stupid, but that's how it usually comes across to others. So, you always have these prejudices when you hear that. I think the word is the problem. Personally, I've never had such a big prejudice, but it's always so difficult for others. But I would say that it has changed for me.*” (Interviewee B). “*The more obese you are or the more you don't like yourself with the weight you have and the way you look, the shyer you are or the more you isolate yourself and the more vulnerable you are to some proles who think they have to reduce you down to your looks or weight. And that wasn't the case here.*” (Interviewee E).

###### 3.1.4.1.3 Subtheme 10.3. Previous therapy experience

Most participants had limited prior therapy experience and either did not know what to expect from the intervention or had negative expectations. They all reported that the intervention was better than expected. Moreover, participants reported that they would be open to going into treatment again because of the positive experiences they had with DIGIT.

## 4 Discussion

The present study explored participants´ experience, perceived benefits, and acceptability of the DIGIT treatment using the qualitative approach of the reflexive thematic analysis. Our qualitative findings resonate with Krakowczyk et al. (2024), who reported that participants' perceptions of usefulness and trust in online mental-health materials strongly influenced their engagement. As a result of the analysis of the interview data, participants showed high satisfaction and acceptance of DIGIT. They evaluated it as a supportive intervention, making them subjectively more active and positive, as well as enabling better disease-related coping.

The category “user experience” was clustered into four themes, namely interpersonal contact, digital vs hybrid vs face-to-face, the technical framework, and DIGIT as an integrated treatment concept, with five additional subthemes. Overall, all participants reported a positive attitude and high acceptance of DIGIT. The acceptance of e-health applications is connected to higher adherence and, therefore, important for the subsequent effectiveness (Rój, 2022). Previous studies have shown a moderate to high acceptance of eHealth interventions for disease management (Bäuerle et al., 2022; Schröder et al., 2023). The hybrid approach added the element of in-person sessions and was associated with high reported acceptance among participants. Surprisingly, all participants, despite various age ranges being included, showed a high acceptance. Nonetheless, previous research showed that there might be an age effect, indicating that younger individuals show a higher level of acceptance towards eHealth applications (Bäuerle et al., 2022). Hybrid interventions might lower the threshold to introduce individuals of various demographics to digital applications.

Participants experienced an accepting, tolerant, and judgment-free space within the patient group and with the therapists. Shared experiences among group members resulted in a sense of connectedness and community, which is common in group therapy (Bledin, 2022). The one-on-one sessions were described as a space to discuss issues that could not be discussed elsewhere because of fear of stigma and not being understood. The reduction of self-stigma and stigma from others can help to moderate weight loss, as well as promote the quality of life of patients (Lillis et al., 2019).

Moreover, patients drew a comparison to the mandatory weight loss surgery preparation program in Germany, for which they had to attend six sessions of nutritional consultations. The nutritional researcher was described as more competent and facts-oriented, which is an important factor for the outcome expectancy of interventions and ultimately the effectiveness (Rój, 2022). The individual psychotherapy sessions were evaluated as necessary to discuss strategies to deal with distress before surgery, which is not accounted for in the surgery program (Johnston et al., 2023).

Compared to self-help groups, individuals positively evaluated the professionally guided group sessions. The participants all acknowledged the need to address psychological components of weight gain, such as internal and external factors that can lead to emotional eating and unhealthy lifestyle outcomes. This is in line with the biopsychosocial model, which can explain the various factors that contribute to the development and maintenance of obesity (Rosenbaum and White, 2016).

The predominantly digital implementation of DIGIT was evaluated as positive and highly accepted, even though some participants enjoyed the combination of onsite and online treatment (hybrid). Previous research has shown that blended psychotherapy, referring to the combination of digital and face-to-face psychotherapy, has various advantages compared to onsite therapy alone, such as being more flexible. Compared to online therapy alone, hybrid formats can heighten the patient's adherence, decrease dropout rates, and are a valuable tool in crisis intervention (Bielinski et al., 2021).

The participants all encountered technical difficulties with the platform for video consultations, which is one disadvantage of online therapy, and they underlined the importance of open communication and catch-up meetings. Only one participant raised concerns about data protection but felt safe during the intervention. This is not in line with other studies, which identified data protection as a major barrier to e-health applications (Alhammad et al., 2024; Nicholas et al., 2017). Therefore, data protection should be communicated openly and considered when implementing eHealth and telehealth interventions in the future.

The second category (Participant Needs) includes the single theme, Recommendations for the intervention. Altogether, participants expressed high satisfaction and acceptance of DIGIT and only had a few recommendations. The design of the intervention, with weekly individual and group therapy and a website with digital worksheets, was rated as complementary, and no parts were missed by participants.

The interprofessional nature of DIGIT is based on research on obesity management programs, which underline the advantages, including higher satisfaction, increased quality of life, weight loss, and lower distress (Eliot et al., 2021; Kouidrat et al., 2024). As for the recommendations for DIGIT, participants would have liked to continue with DIGIT and therefore recommend a longer treatment duration. As some participants prepared for weight loss surgery, specific content was recommended, which was only discussed in the individual meetings instead of the group sessions, because of the heterogeneous nature of the group. In addition, participants emphasized the importance of increasing the visibility and awareness of DIGIT to make the program accessible to a wider group of patients. Furthermore, many participants expressed a desire for an aftercare component, such as follow-up sessions, in which they could reflect on their progress and continue to receive support after completing the intervention.

The third category (Perceived benefits) was in line with the anticipated aim of DIGIT. All participants perceived the DIGIT Intervention as beneficial and reported becoming more active, positive, and developing better disease-related coping. All individuals introduced new routines into their everyday lives and adaptive coping strategies (e.g., cooking, improved sleep–wake pattern, journaling, sports). The intricate link between mood and food choices can partly explain why obesity and mental disorders, especially depression, are associated. Moreover, obesity is often associated with decreased activity. Therefore, focusing on dietary, emotional, and environmental factors as a personalized approach is necessary to form long-term, maintainable habits (Calcaterra et al., 2024; Koski and Naukkarinen, 2017).

Moreover, all participants would recommend the intervention to other individuals with obesity and distress. Participants also reported that positive changes were noticed in their social systems, possibly underlining changes perceived by participants as related to DIGIT. The effectiveness of mindfulness and acceptance-based therapy is in line with the literature, which underlines the effectiveness for bodily distress, depression, and anxiety disorders (Maas genannt Bermpohl et al., 2023). Participants preparing for weight loss surgery also reported a stabilization before surgery, which was accompanied by subjectively lower levels of anxiety. Studies have shown that a lower preoperative anxiety level is one of the few predictors of long-term weight loss after surgery (Lüscher et al., 2023). DIGIT could therefore potentially be a useful intervention for supporting a subgroup of individuals undergoing bariatric surgery who experience higher levels of anxiety. Whether DIGIT influences postoperative outcomes, including long-term weight loss, cannot be determined from the present qualitative data and should therefore be investigated in future studies.

There is a multitude of barriers that hinder individuals with obesity from receiving adequate healthcare. These comprise physical immobility, financial struggles, and mental comorbidities, which are often associated with social withdrawal. As well as external barriers, such as the geographic location and shortage of healthcare professionals (Geiger et al., 2023; Washington et al., 2023). Moreover, societal barriers such as the stigma of seeking mental health services impede individuals from receiving adequate support (Puhl and Heuer, 2010; Steubl and Baumeister, 2023). The fourth category (Facilitated access to treatment) focused on factors that reduce barriers to on-site inpatient and outpatient therapy. The first identified subtheme was the flexibility of time and place. Participants reported that they were able to integrate DIGIT into their daily lives by not having to travel to the on-site facility and by being able to schedule a personal meeting time that was compatible with their schedules. Compared to inpatient therapy, DIGIT provides in-depth treatment with far fewer resources. It helps individuals stay in their communities and continue working. It removes barriers such as fear of absences at work and inadequate childcare. Thus, the described flexibility of a telehealth interventions like DIGIT might suggest potential resource and productivity benefits (Haleem et al., 2021; Snoswell et al., 2022). Stigmatization of people with obesity and distress was identified as a key issue within the treatment. Ultimately, several factors of stigma were reduced by DIGIT. First, participants reported feeling more comfortable sharing personal information with people who were not close to where they lived. Second, most participants had not been in therapy before and reported that their attitudes and prejudices about the word therapy had changed and that they would be more open with their social systems. Research has shown that contact with other people with similar symptoms is the most effective strategy for reducing stigma by promoting real-life examples and thereby overcoming unrealistic stereotypes. In addition, education and conversation about stigma can help reduce subsequent stigmatization of self and others (Coman and Sas, 2016; Song et al., 2023). Overall, participants perceived DIGIT as a treatment alternative with a lowered threshold to work on themselves and consider future treatment options.

### 4.1 Limitations

Despite these strengths, the qualitative nature of this study also entails some limitations regarding the generalizability and causal inference of the findings. The sample size of n = 14 is a relatively small and homogeneous sample that may have limited the breadth and diversity of perspectives represented in the analysis (Saunders et al., 2018). Moreover, the participants in this sample resembled a homogenous group, which was characterized as Caucasian, female, and from the western region of Germany. Future or complementary research should therefore include a larger sample size and try to include more diverse individuals (e.g., men, different cultural and ethnic backgrounds) to broaden the perspective. The study was conducted in a single-center setting and included a completer-only sample, which may limit the generalizability of the findings and potentially introduce selection bias. Further research using multicenter designs and intention-to-treat analyses is warranted to confirm these findings. Moreover, the recruitment of participants could lead to a selection bias since they had to be willing to participate in the intervention and therefore might already be more open and positive towards it. Even though participants were encouraged to disclose negative aspects of the intervention, socially desirable answers cannot be ruled out and are likely to occur when evaluating a therapeutic intervention (Bispo Júnior, 2022; Collier and Mahoney, 1996).

Integrating qualitative findings into a mixed-methods approach may provide a more holistic understanding of the underlying mechanisms while allowing for better adaptation to patients' needs and demands (Bartholomew and Lockard, 2018). Further research could also focus on a quantitative approach to analyze if hybrid interventions for obesity and mental health burden also result in objective measures of associated symptom relief. Moreover, factors associated with specific sub-samples of the intervention, such as post-operative factors (e.g., adherence factors, weight loss), could be researched. In addition, different blended care formats could be introduced, such as an app design that provides additional personalized treatment content and could be compared to treatment as usual.

## 5 Conclusion

The innovative intervention DIGIT for individuals with obesity and comorbid distress was described as highly acceptable. Patients reported positive changes in self-care, social interactions, positive self-perception, and the integration of new routines in everyday life. Moreover, an overarching ‘activating effect’ was identified. This study highlights potential advantages of digital inpatient-like therapy concepts. Patients' high acceptance of and satisfaction with the inpatient-like therapy DIGIT is an important facilitator for the future implementation of innovative treatment concepts. Treatment concepts like DIGIT can contribute to providing individualized therapy options and closing care gaps. As individuals with obesity and comorbid psychological distress face a multitude of barriers in conventional therapy offerings, inpatient-like therapy concepts like DIGIT could ultimately help surmount these by introducing a low-threshold, flexible, and potentially resource-saving treatment and making healthcare more accessible.

## CRediT authorship contribution statement

**Tania Lalgi:** Conceptualization, Methodology, Investigation, Validation, Formal analysis, Data curation, Writing – original draft. **Charlotte Michel:** Conceptualization, Methodology, Investigation, Validation, Formal analysis, Data curation, Writing – original draft. **Sophie Schulz Genannt Menningmann:** Conceptualization, Methodology, Investigation, Writing – review & editing. **Britta Pehlke:** Conceptualization, Project administration, Writing – review & editing, Supervision. **Christoph Jansen:** Conceptualization, Project administration, Writing – review & editing, Supervision. **Till Hasenberg:** Conceptualization, Project administration, Writing – review & editing, Supervision. **Martin Teufel:** Conceptualization, Project administration, Writing – review & editing, Supervision. **Alexander Bäuerle:** Conceptualization, Project administration, Writing – review & editing, Supervision. **Anita Robitzsch:** Conceptualization, Project administration, Writing – review & editing, Supervision.

## Funding

No external funding was received for the study or the development of DIGIT. The Open Access Fund of the University of Duisburg-Essen supported publication costs.

## Declaration of competing interest

None declared.

## Data Availability

The data supporting the present results are not publicly available due to privacy concerns and the risk of participant re-identification. Anonymized data may be available from the corresponding author upon reasonable request, subject to applicable ethical and data protection requirements.

## References

[bb0005] Alhammad N., Alajlani M., Abd-Alrazaq A., Epiphaniou G., Arvanitis T. (2024). Patients’ perspectives on the data confidentiality, privacy, and security of mHealth apps: systematic review. J. Med. Internet Res..

[bb0010] audiotranskription (2024). Automatische Transkription mit f4 [Computer Software]. https://www.audiotranskription.de/f4x/.

[bb0015] Bartholomew T.T., Lockard A.J. (2018). Mixed methods in psychotherapy research: a review of method(ology) integration in psychotherapy science. J. Clin. Psychol..

[bb0020] Bäuerle A., Frewer A.-L., Rentrop V., Schüren L.C., Niedergethmann M., Lortz J., Skoda E.-M., Teufel M. (2022). Determinants of acceptance of weight management applications in overweight and obese individuals: using an extended unified theory of acceptance and use of technology model. Nutrients.

[bb0025] Bielinski L.L., Trimpop L., Berger T. (2021). Die Mischung macht’s eben? Blended-Psychotherapie als Ansatz der Digitalisierung in der Psychotherapie [All in the mix? Blended psychotherapy as an example of digitalization in psychotherapy]. Psychotherapeut.

[bb0030] Bispo Júnior J.P. (2022). Social desirability bias in qualitative health research. Rev. Saude Publ..

[bb0035] Bledin K. (2022). Book review: the theory and practice of group psychotherapy. Group Anal..

[bb0040] Braun V., Clarke V. (2006). Using thematic analysis in psychology. Qual. Res. Psychol..

[bb0045] Braun V., Clarke V. (2023). Toward good practice in thematic analysis: avoiding common problems and be(com)ing a knowing researcher. Int. J. Transgender Health.

[bb0050] Brown W., Yen P.-Y., Rojas M., Schnall R. (2013). Assessment of the health IT usability evaluation model (health-ITUEM) for evaluating mobile health (mHealth) technology. J. Biomed. Inform..

[bb0055] Bryman A., Bell E., Teevan J.J. (2012).

[bb0060] Calcaterra V., Rossi V., Magenes V.C., Baldassarre P., Grazi R., Loiodice M., Fabiano V., Zuccotti G. (2024). Dietary habits, depression and obesity: an intricate relationship to explore in pediatric preventive strategies. Front. Pediatr..

[bb0065] Castelnuovo G., Pietrabissa G., Manzoni G.M., Cattivelli R., Rossi A., Novelli M., Varallo G., Molinari E. (2017). Cognitive behavioral therapy to aid weight loss in obese patients: current perspectives. Psychol. Res. Behav. Manag..

[bb0070] Collier D., Mahoney J. (1996). Insights and pitfalls: selection bias in qualitative research. World Polit..

[bb0075] Coman A., Sas C. (2016).

[bb0080] Creswell J.W., Hanson W.E., Clark Plano V.L., Morales A. (2007). Qualitative research designs. Couns. Psychol..

[bb0085] Dandgey S., Patten E. (2023). Psychological considerations for the holistic management of obesity. Clin. Med. (Lond.).

[bb0090] Eliot K., Cuff P., Firnhaber G., Kolasa K.M. (2021). Interprofessional obesity treatment: an exploration of current literature and practice. J. Interprofessional Educ. Pract..

[bb0095] Elliott R., Slatick E., Urman M. (2001). Qualitative change process research on psychotherapy: alternative strategies. Psychol. Test Assess. Model..

[bb0100] Fereday J., Muir-Cochrane E. (2006). Demonstrating rigor using thematic analysis: a hybrid approach of inductive and deductive coding and theme development. Int. J. Qual. Methods.

[bb0105] Galin B. (2024). Implementing a hybrid model of therapy is a win for therapists, patients and payers. Int. J. Sports Phys. Ther..

[bb0110] Geiger S., Steinbach J., Skoda E.-M., Jahre L.M., Rentrop V., Kocol D., Jansen C., Schüren L., Niedergethmann M., Teufel M., Bäuerle A. (2023). Needs and demands for E-mental health interventions in individuals with overweight and obesity: user-centred design approach. Obes. Facts.

[bb0115] Haleem A., Javaid M., Singh R.P., Suman R. (2021). Telemedicine for healthcare: capabilities, features, barriers, and applications. Sensors Int..

[bb0120] Healthy Projects GmbH (2026).

[bb0125] Johnston L., Jackson K., Hilton C., Graham Y. (2023). The forgotten patient: a psychological perspective on the implementation of bariatric surgery guidelines. Obes. Sci. Pract..

[bb0130] Kocol D., Bäuerle A., Schadendorf T., Geiger S., Krakowczyk J.B., Skoda E.-M., Teufel M. (2024). Efficacy of eHealth interventions to reduce depression symptoms in individuals with obesity: a systematic review of randomized controlled trials. Front. Psychol..

[bb0135] Koliaki C., Dalamaga M., Liatis S. (2023). Update on the obesity epidemic: after the sudden rise, is the upward trajectory beginning to flatten?. Curr. Obes. Rep..

[bb0140] Koski M., Naukkarinen H. (2017). Severe obesity, emotions and eating habits: a case-control study. BMC Obes..

[bb0145] Kouidrat Y., Louhou R., Mondot C., Daami I., Amad A., Diouf M. (2024). Quality of life in patients with obesity: the role of multidisciplinary rehabilitation medicine. Obesities.

[bb0150] Krakowczyk J.B., Truijens F.L., Teufel M., Lalgi T., Heinen J., Schug C., Erim Y., Pantförder M., Graf J., Bäuerle A. (2024). Evaluation of the E-mental health intervention make it training from patients’ perspectives: qualitative analysis within the reduct trial. JMIR Cancer.

[bb0155] Kupila S.K.E., Joki A., Suojanen L.U. (2023). The effectiveness of eHealth interventions for weight loss and weight loss maintenance in adults with overweight or obesity: a systematic review of systematic reviews. Curr. Obes. Rep..

[bb0160] Lillis J., Thomas J.G., Olson K., Wing R.R. (2019). Weight self-stigma and weight loss during behavioural weight loss intervention. Obes. Sci. Pract..

[bb0165] Lüscher A., Vionnet N., Amiguet M., Chartoumpekis D., Mantziari S., Frantz J., Favre L. (2023). Impact of preoperative psychiatric profile in bariatric surgery on long-term weight outcome. Obes. Surg..

[bb0170] Lutz W., Hill C.E. (2009). Quantitative and qualitative methods for psychotherapy research: introduction to special section. Psychother. Res..

[bb0175] Maas genannt Bermpohl F., Hülsmann L., Martin A. (2023). Efficacy of mindfulness- and acceptance-based cognitive-behavioral therapies for bodily distress in adults: a meta-analysis. Front. Psychol..

[bb0180] Myers M.D., Newman M. (2007). The qualitative interview in IS research: examining the craft. Inf. Organ..

[bb0185] Nicholas J., Huckvale K., Larsen M.E., Basu A., Batterham P.J., Shaw F., Sendi S. (2017). Issues for eHealth in psychiatry: results of an expert survey. J. Med. Internet Res..

[bb0190] Puhl R.M., Heuer C.A. (2010). Obesity stigma: important considerations for public health. Am. J. Public Health.

[bb0195] Rajan T.M., Menon V. (2017). Psychiatric disorders and obesity: a review of association studies. J. Postgrad. Med..

[bb0200] Rentrop V., Damerau M., Schweda A., Steinbach J., Schüren L.C., Niedergethmann M., Skoda E.-M., Teufel M., Bäuerle A. (2022). Predicting acceptance of E-mental health interventions in patients with obesity by using an extended unified theory of acceptance model: cross-sectional study. JMIR Form. Res..

[bb0205] Rój J. (2022). What determines the acceptance and use of eHealth by older adults in Poland?. Int. J. Environ. Res. Public Health.

[bb0210] Rosenbaum D.L., White K.S. (2016). Understanding the complexity of biopsychosocial factors in the public health epidemic of overweight and obesity. Health Psychol. Open.

[bb0215] samedi GmbH (2026). Samedi Videosprechstunde (Video consultation platform). https://video.samedi.de/.

[bb0220] Sarwer D.B., Polonsky H.M. (2016). The psychosocial burden of obesity. Endocrinol. Metab. Clin. N. Am..

[bb0225] Saunders B., Sim J., Kingstone T., Baker S., Waterfield J., Bartlam B., Burroughs H., Jinks C. (2018). Saturation in qualitative research: exploring its conceptualization and operationalization. Qual. Quant..

[bb0230] Schmidt-Hantke J., Vollert B., Hagner F., Beintner I., Hütter K., Nitsch M., Jacobi C., Waldherr K. (2021). Stakeholders’ perspectives on online interventions to improve mental health in eating disorder patients and carers in Germany. Eur. J. Pub. Health.

[bb0235] Schröder J., Bäuerle A., Jahre L.M., Skoda E.-M., Stettner M., Kleinschnitz C., Teufel M., Dinse H. (2023). Acceptance, drivers, and barriers to use eHealth interventions in patients with post-COVID-19 syndrome for management of post-COVID-19 symptoms: a cross-sectional study. Ther. Adv. Neurol. Disord..

[bb0240] Snoswell C.L., Smith A.C., Page M., Scuffham P., Caffery L.J. (2022). Quantifying the societal benefits from telehealth: productivity and reduced travel. Val. Health Reg. Iss..

[bb0245] Song N., Hugh-Jones S., West R.M., Pickavance J., Mir G. (2023). The effectiveness of anti-stigma interventions for reducing mental health stigma in young people: a systematic review and meta-analysis. Glob. Mental Health.

[bb0250] Steubl L.S., Baumeister H. (2023). Videobasierte Psychotherapie: Aktuelle Rahmenbedingungen und Entwicklungen sowie Empfehlungen für die praktische Umsetzung [Video-based psychotherapy: current framework conditions, developments, and recommendations for practical implementation]. Bundesgesundheitsbl. Gesundheitsforsch. Gesundheitsschutz.

[bb0255] Tong A., Sainsbury P., Craig J. (2007). Consolidated criteria for reporting qualitative research (COREQ): a 32-item checklist for interviews and focus groups. Int. J. Qual. Health Care J. Int. Soc. Qual. Health Care.

[bb0260] Truijens F.L. (2017). Do the numbers speak for themselves? A critical analysis of procedural objectivity in psychotherapeutic efficacy research. Synthese.

[bb0265] VERBI Software (2021). MAXQDA 2022 [Computer Software]. https://www.maxqda.com/de/software-inhaltsanalyse.

[bb0270] Washington T.B., Johnson V.R., Kendrick K., Ibrahim A.A., Tu L., Sun K., Stanford F.C. (2023). Disparities in access and quality of obesity care. Gastroenterol. Clin. N. Am..

[bb0275] Wilmers F., Munder T., Leonhart R., Herzog T., Plassmann R., Barth J., Linster H.W. (2008). Die deutschsprachige Version des Working Alliance Inventory-short revised (WAI-SR) — Ein schulenübergreifendes, ökonomisches und empirisch validiertes Instrument zur Erfassung der therapeutischen Allianz. Klin. Diagn. Eval..

[bb0280] World Health Organization (2024). Obesity and Overweight. https://www.who.int/news-room/fact-sheets/detail/obesity-and-overweight.

[bb0285] Yardley L. (2000). Dilemmas in qualitative health research. Psychol. Health.

