# Supplementary material for: Participants' experiences with the hybrid digital psychotherapy intervention-DIGIT for obesity and distress: A qualitative interview study
Source: Internet Interv. 2026 Sep 8;46:100997. doi: 10.1016/j.invent.2026.100997 (PMC13584009; doi:10.1016/j.invent.2026.100997)
Supplement: Supplementary file 1 [file mmc1.docx]

# **Appendix**

**Table A1**

*Consolidated criteria for reporting qualitative research (COREQ; [1])*

| **Domain 1: Research team and reflexivity**  *Personal characteristics* | | **Page** |
| --- | --- | --- |
| I. Facilitator | Tania Lalgi; Charlotte Michel; Sophie Schulz genannt Menningmann; Britta Pehlke, Till Hasenberg; Christoph Jansen; Martin Teufel; Alexander Bäuerle; Anita Robitzsch | 1 |
| II. Credentials | Tania Lalgi (M.Sc.)  Charlotte Michel (M.Sc.)  Sophie Schulz genannt Menningmann (B.Sc.)  Britta Pehlke (PhD)  Till Hasenberg (MD)  Christoph Jansen (CJ)  Martin Teufel (MD)  Alexander Bäuerle (PhD)  Anita Robitzsch (MD) |  |
| III. Occupation | CM, TL, SSGM, CJ: research associate  AB, BP, AR: postdoctoral researcher  TH, MT: head of department, full-time professor | 4-7 |
| IV. Gender | Woman: TL, CM, SSGM, BP, AR  Man: TH, CJ, MT, AB |  |
| V. Experience and training | CM: educational background in psychology (M.Sc.)  TL: educational background in psychology (M.Sc.), doctoral candidate  SSGM: educational background in psychology (B.Sc.)  BP: educational background in psychology (M.Sc.), PhD, postdoctoral researcher, and co-head of the research group obesity and mental health burden  AB: educational background in psychology (M.Sc.), postdoctoral researcher and head of research  TH: physician and specialized in surgery, head of department  CJ: physician and specialized in psychosomatic medicine and psychotherapy, head of inpatient unit and head of the research group Development and Programming  MT: physician and specialized in psychosomatic medicine and psychotherapy, head of department  AR: physician and specialized in psychosomatic medicine and psychotherapy, head of outpatient clinic and head of the research group obesity and mental health burden |  |
| ***Relationship with participants*** | |  |
| VI. Relationship established | The Participants in the present study had no prior relationship established to the facilitator | 6-7 |
| VII. Participant knowledge of facilitator | The interviewer was not part of the DIGIT treatment team and had no therapeutic relationship with participants | 7-8 |
| VIII. Facilitator characteristics | No other characteristics were reported about the facilitator |  |

| **Domain 2: Study design** | |
| --- | --- |
| ***Theoretical framework*** | |
| Methodological orientation and theory | Reflexive thematic analysis by Braun and Clarke (2006), using a combined deductive–inductive approach. |
| ***Participant selection*** | |
| Sampling | Consecutive sampling of eligible patients from the outpatient obesity consultation service. Recruiting from 01-01-2024 to 28-02-2024 |
| Method of approach | Recruited face-to-face during initial consultations at the obesity outpatient clinic of the Department of Psychosomatic Medicine and Psychotherapy, LVR-University Hospital Essen |
| Sample size | *N* = 14 participants |
| Non-participation | At the qualitative interview stage, none: all 14 eligible DIGIT completers agreed to participate. |
| ***Setting*** | |
| Setting of data collection | Digitally and face-to-face |
| Presence of non-participants | No presence of non-participants. |
| Description of sample | Fourteen adults with severe obesity and self-reported psychological distress participated. Thirteen participants were women and one was a man; mean age was 48.4 years. Further sociodemographic and clinical characteristics are reported in Table 1. |
| ***Data collection*** | |
| Interview guide | Provided as supplemental material |
| Repeat interviews | None |
| Audio/visual recording | Audio recording |
| Field notes | No |
| Duration | 15 to 32 mins |
| Data saturation | Data saturation was not used as a formal stopping criterion. The qualitative sample comprised all eligible participants who completed DIGIT and participated in the subsequent interview. |
| Transcripts returned | No |

| **Domain 3: Analysis and findings** |  |
| --- | --- |
| Number of data coders | Initial coding by CM and SSGM. A third researcher (TL) contributed to the iterative development and refinement of themes. |
| Description of coding tree | Yes (in the results section in Figure 2) |
| Derivation of themes | (1) user experience (2) *participant´ needs* (3) *perceived benefits* (4) facilitated access to treatment |
| Software | MaxQDA 2022 |
| Participant checking | No; transcripts or preliminary themes were not returned to participants for member checking. |
| ***Reporting*** | |
| Quotations presented | Yes |
| Data and findings consistent | Yes |
| Clarity of major themes | Yes |
| Clarity of minor themes | Yes |

**Table A2**

*Semi-structured interview guideline*

| Segments | Questions | Source |
| --- | --- | --- |
| Attitudes towards digital healthcare applications | 1. What are your general impressions and experiences with the use of digital technologies and media in healthcare or in the context of your medical healthcare (e.g., e-prescription, digital appointment booking, digital referral, virtual consultation)? 2. What factors would make it easier for you to continue or resume participating in digitalized treatment? | Self-constructed |
| General experience of the intervention | 3. What is your general opinion of the implementation of the Digital Clinic in terms of content? 4. What do you generally think of the technical implementation of the Digital Clinic? | Based on Client Change Interview  (Elliott et al., 2001) |
| Expectations of the intervention | 5. What expectations did you have when you started the Digital Clinic? 6. To what extent have your expectations been confirmed? What was different? | Self-constructed |
| (hybrid) relationship to group | 7. To what extent did you feel connected to other patients? (e.g., personal exchange, mutual understanding, sense of community) | Self-constructed |
| (Hybrid) relationship to therapist | 8. To what extent did you feel connected to the therapists? (e.g., understanding, sense of community) 9. To what extent did you feel that you were working together with your therapists towards therapy goals? *(Therapy goals)* 10. To what extent does the therapy give you a new perspective on possible problems? *(Agreement on therapeutic approach)* 11. To what extent do you feel that your therapist appreciates you? *(Attachment)* | (Based on the WAI-SR Questionnaire – Working Alliance Inventory)  (Wilmers et al., 2008) |
| Perceived changes and helpful aspects | 12. Are there any changes in you that you have noticed since our treatment? | Based on Client Change Interview (Elliott et al., 2001) |
| Perceived changes and helpful aspects | 13. If I were to ask someone close to you what has changed during the 6-week period of digital treatment, what would they tell me? (e.g., family, friends, employer) | Self-constructed |
| Changes perceived by social environment | 14. What do you think caused these changes? Which aspect/content was the most helpful? 15. Were there any aspects/contents of the digital treatment that you found helpful? What do you think it was because of? 16. Which aspect/content was the most helpful? | Based on Client Change Interview  (Elliott et al., 2001) |
| Perceived changes and helpful aspects | 17. What added value do you take away from the digital clinic? (e.g., routines, habits, changed eating behavior, food choices, etc.) | Self-constructed |
| Missing aspects/ content & hurdles | 18. Were there any aspects/contents of the digital clinic that were unhelpful, hindering, negative or disappointing for you? (e.g., poor internet connection or less personal through video therapy, difficulties building trust and group dynamics)? - own examples used 19. What made these aspects unhelpful? 20. In your opinion, what content was neglected or missing in the Digital Clinic? | Based on Client Change Interview  (Elliott et al., 2001) |
| Motivation | 21. How easy was it for you to motivate yourself to participate in the Digital Clinic? (Both individual and group sessions) | Based on Client Change Interview  (Elliott et al., 2001) |
| Usability | 22. To what extent do you think the Digital Clinic could improve the quality of life for individuals with obesity and psychological distress? | Based on the Health-Information Technology Usability Survey (Health ITUES) (Brown et al., 2013) |
| Recommendations | 23. If a friend needed similar help, would you recommend the Digital Clinic to him or her? | Based on Client Change Interview  (Elliott et al., 2001) |
|  | 24. To what extent do you think the Digital Clinic could improve the quality of life for patients with obesity? | Based on Health-Information Technology Usability Survey (Health ITUES) (Brown et al., 2013) |
| Summary (best vs. worse) | 25. What are the three best aspects of the digital clinic? 26. What are the three most negative aspects of the Digital Clinic? | Self-constructed |

**Table A3**

*Session-by-session structure and content of the six-week DIGIT intervention*

|  | **Group therapy**  (75 min) | **Individual session**  (50 min) |
| --- | --- | --- |
| **Week 1**  Title: *Kick-off* | Introduction – theoretical background on the connection between obesity and psychological well-being, establishment of group rules, intervention structure, expectations & concerns, emergency procedures & contacts | Psychotherapy:  Understanding the bidirectional relationship between obesity and psychological distress, goal setting |
| Digital worksheets | (1) Questionnaire on Contributing Factors (obesity & psychological distress)  (2) Personal change goals worksheet | (1) Individual development and maintenance model |
| **Week 2**  Title:  *Emotions & mindfulness* | Introduction to theories of emotions & emotional regulation techniques, mindfulness, emotional eating & individual experiences of patients | Nutritional therapy:  Dietary assessment: current and desired dietary patterns |
| Digital worksheets | (1) Emotion monitoring and situation analysis worksheet | (1) Dietary assessment  (2) Dietary intake diary |
| **Week 3**  Title:  *Influencing factors* | Weight stigma and connection to obesity and psychological distress, everyday-life impact; positive and negative influencing factors & individual agency | Psychotherapy:  ABC model of cognitive-behavioural analysis – situational analyses based on the individual development and maintenance model from session 1 |
| Digital worksheets | (1) Individual influencing factors: What affects my daily life, obesity, and psychological distress?  (2) promoting positive influences | (1) ABC model worksheet |
| **Week 4**  Title:  *Life 2.0* | Future perspectives: post-weight-loss/post-bariatric-surgery life, psychological and physical changes, realistic goals;  physical activity & breathing exercises  (Session held by a psychologist & an exercise therapist) | Nutritional therapy:  Dietary diary review and individualized counselling based on goals established in Session 1, acknowledgment and reinforcement of positive behaviours |
| Digital worksheets | (1) Self-Care Activities Worksheet  (2) Physical Activity: What Do I Enjoy? | / |
| **Week 5**  Title:  *Emergency toolkit* | Coping skills; self-compassion, behavioural strategies in challenging situations, individual resources | Psychotherapy:  Personal coping strategies, progress review, and relapse prevention |
| Digital worksheets | (1) My individual emergency coping toolkit | / |
| ***Week 6***  Title:  *Outlook & relapse prevention* | Review; consolidation; progress; maintenance strategies; relapse prevention; future planning  (No worksheets) | Nutritional therapy:  Review of progress and consolidation of individual nutrition goals  (No worksheets) |
